# Identification and evolution of ICE-*Pmu*ST394: a novel integrative conjugative element in *Pasteurella multocida* ST394

**DOI:** 10.1093/jac/dkae040

**Published:** 2024-02-21

**Authors:** Piklu Roy Chowdhury, Tamara Alhamami, Henrietta Venter, Tania Veltman, Mandi Carr, Joanne Mollinger, Darren J Trott, Steven P Djordjevic

**Affiliations:** Australian Institute for Microbiology and Infection, University of Technology Sydney, City Campus, Ultimo, NSW 2007, Australia; Australian Centre for Antimicrobial Resistance Ecology, School of Animal and Veterinary Sciences, University of Adelaide, Roseworthy Campus, Roseworthy, SA 5371, Australia; Clinical Health Sciences, University of South Australia, Adelaide, SA 5000, Australia; Australian Centre for Antimicrobial Resistance Ecology, School of Animal and Veterinary Sciences, University of Adelaide, Roseworthy Campus, Roseworthy, SA 5371, Australia; Australian Centre for Antimicrobial Resistance Ecology, School of Animal and Veterinary Sciences, University of Adelaide, Roseworthy Campus, Roseworthy, SA 5371, Australia; Biosecurity Sciences Laboratory, Department of Agriculture and Fisheries, Health and Food Sciences Precinct, Coopers Plains, QLD 4108, Australia; Australian Centre for Antimicrobial Resistance Ecology, School of Animal and Veterinary Sciences, University of Adelaide, Roseworthy Campus, Roseworthy, SA 5371, Australia; Australian Institute for Microbiology and Infection, University of Technology Sydney, City Campus, Ultimo, NSW 2007, Australia

## Abstract

**Background:**

The emergence of macrolide and tetracycline resistance within *Pasteurella multocida* isolated from feedlot cattle and the dominance of ST394 in Australia was reported recently.

**Objectives:**

To establish the genetic context of the resistance genes in *P. multocida* 17BRD-035, the ST394 reference genome, and conduct a molecular risk assessment of their ability to disperse laterally.

**Methods:**

A bioinformatic analysis of the *P. multocida* 17BRD-035 genome was conducted to determine if integrative conjugative elements (ICEs) carrying resistance genes, which hamper antibiotic treatment options locally, are in circulation in Australian feedlots.

**Results:**

A novel element, ICE-*Pmu*ST394, was characterized in *P. multocida* 17BRD-035. It was also identified in three other isolates (two ST394s and a ST125) in Australia and is likely present in a genome representing *P. multocida* ST79 from the USA. ICE-*Pmu*ST394 houses a resistance module carrying two variants of the *bla*_ROB_ gene, *bla*_ROB-1_ and *bla*_ROB-13_, and the macrolide esterase gene, *estT*. The resistance gene combination on ICE-*Pmu*ST394 confers resistance to ampicillin and tilmicosin, but not to tulathromycin and tildipirosin. Our analysis suggests that ICE-*Pmu*ST394 is circulating both by clonal expansion and horizontal transfer but is currently restricted to a single feedlot in Australia.

**Conclusions:**

ICE-*Pmu*ST394 carries a limited number of unusual antimicrobial resistance genes but has hotspots that facilitate genomic recombination. The element is therefore amenable to hosting more resistance genes, and therefore its presence (or dispersal) should be regularly monitored. The element has a unique molecular marker, which could be exploited for genomic surveillance purposes locally and globally.

## Introduction

Bovine respiratory disease (BRD) is the most common disease in farmed cattle, resulting in significant adverse effects on the economy and necessitating careful monitoring and prevention of drug-resistant infections locally and globally.^1,2^ In the recent past, North America has seen a rapid rise in resistant variants of the two most predominant BRD pathogens, *Pasteurella multocida* and *Mannheimia haemolytica*, resulting in infections resistant to first- and second-line antibiotics prescribed for BRD.^3–7^ Acquisition of integrative conjugative elements (ICEs) was linked to this rapid upsurge of the resistance problem in North America^8–10^ and Europe;^11^ however, such elements are yet to be reported in Australia.

According to Meat and Livestock Australia estimates, Australia produced 2.4 million tonnes of beef and veal, of which 76% was exported at an estimated return of A$10.8 bn^12^ in 2019. Although prudent use of antibiotics for therapeutic and metaphylactic purposes in feedlot cattle has prevented *de novo* appearance of resistance locally; we have identified subsets of *P. multocida* isolates that were resistant to various drug combinations of tetracycline, tilmicosin, tulathromycin/gamithromycin and ampicillin/penicillin,^13^ including an unusual phenotype of ampicillin/penicillin, tetracycline and tilmicosin^13^ resistance but susceptibility to tulathromycin. Third-generation cephalosporins (ceftiofur), fluoroquinolones (enrofloxacin and danofloxacin), macrolides (gamithromycin, tilmicosin, tildipirosin, tulathromycin), tetracyclines (chlortetracycline, oxytetracycline), florfenicol and sulphonamides are all used to treat BRD internationally.^14^ However, within Australia, fluoroquinolones cannot be used in food-producing animals and third-generation cephalosporins are reserve agents.^15^ Chlortetracycline, oxytetracycline, tilmicosin and tulathromycin remain the most commonly used antimicrobial agents for treating BRD in Australia^16^ as they are rated to be of low importance to human health by the Australian Strategic and Technical Advisory Group on Antimicrobial Resistance.^15^

Our genomic surveillance study on Australian BRD-associated *P. multocida* indicated that ST394 was linked to the emergence of resistance in BRD pathogens.^17^ In 2022, we published the reference genome sequence of *P. multocida* 17BRD-035 (CP082272.1) representing ST394.^18^ Isolate 17BRD-035 was resistant to tetracycline, penicillin and tilmicosin but not tulathromycin.^13^ Sequence analysis revealed the presence of *tet*(R)/*tet*(H) and *bla*_ROB-1_ genes that accounted for tetracycline and penicillin resistance. Genes encoding the MacA-MacB proteins, which constitute a macrolide efflux pump^18^ in *Escherichia coli*, were also identified in the genome. The primary aim of this study was to establish the genetic context of the resistance genes in the genome, with the overarching objective of conducting a molecular risk assessment of the ability of resistance genes to disperse laterally within *P. multocida* and other BRD pathogens in Australia.

## Methods

### Strains, growth conditions and antimicrobial susceptibility testing

Four MDR *P. multocida* (17BRD-035, 18BRD-001, 19BRD-032 and 19BRD-057) representing MLST ST394, isolated from lung swabs of BRD-affected cows in Queensland Australia were re-examined in this study.^13,18^ The strains were grown overnight in brain heart infusion broth for DNA extraction. MIC antimicrobial susceptibility testing was also re-examined and interpreted on the four strains as described previously^13^ using Veterinary Reference Card panels (Sensititre^®^, Trek Diagnostics, Thermo Fisher Scientific, Thebarton, South Australia).

### Genome sequencing

Template DNA for Illumina sequencing was prepared from 4 mL of overnight cultures growing at mid-log phase using the Isolate II Genomic DNA extraction kit (Bioline, Australia) and following the manufacturer’s instructions. Sequencing libraries were prepared from 2 ng of gDNA using the Illumina Nextera XT Library Prep kit following an established protocol.^19^ Multiplexed libraries were sequenced on the Illumina HiSeq platform at the Ramaciotti Centre for Genomics at the University of New South Wales.

For Nanopore sequencing, genomic DNA was prepared using the XS buffer, with modifications.^20^ Briefly, genomic DNA from 2 mL of bacterial cells growing at mid-log phase was resuspended in 1 mL of XS buffer. The cell pellet was lysed by incubation at 70°C, and vortexed briskly to maximize lysis. Cellular debris was precipitated (incubation on ice for 30 min), removed (centrifugation 14 000 rpm, 10 min) and nucleic acid was harvested using an equal volume of isopropanol. Following two 70% ethanol washes, nucleic acid was resuspended gently in 100 µL of TE buffer, RNAse treated (PureLink^™^ RNase A 20 mg/mL from Invitrogen) and DNA was purified using phenol/chloroform extraction. Quality and quantity of DNA was assessed using Nanodrop and Qbit prior to the preparation of sequencing libraries. Multiplexed sequencing libraries were prepared using Oxford Nanopore Technologies’ (ONT’s) Rapid Barcoding Sequencing Kit (SQK-RBK004) with 500 ng of input genomic DNA per sample and sequenced using a R9.4.1 flow cell (FLO-MIN106) at the DNA sequencing facility of the Australian Institute for Microbiology and Infection at UTS.

### Assembly of genome sequences

FastQC (https://www.bioinformatics.babraham.ac.uk/projects/fastqc/) was used to assess the quality of Illumina sequences. Guppy base caller was used to extract long-read sequences from the multiplexed Nanopore run and demultiplexed using Deepbinner.^21^ FastQC, FastP^22^ and pycoQC^23^ were used for quality control purposes and MultiQC^24^ was used to collate QC data into a single file. Genomes were co-assembled using Unicycler^25^ and can be accessed in GenBank using nucleotide accession numbers JARXZF000000000.1, JARXZE000000000.1 and CP123618.1 for 18BRD-001, 19BRD-032 and 19BRD-057, respectively.

### Sequence analysis and annotation

Preliminary genome annotations were generated using Prokka^26^ and on the RAST server.^27^ The ICEberg 2.0^28^ database was used to identify the location of ICEs and integrative mobilizable elements (IMEs) and the annotations were manipulated using SnapGene (https://www.snapgene.com/). Individual modules on the ICE were manually curated using iterative BLASTn and BLASTp^29^ searches. The allelic variants of the *bla*_ROB_ genes were confirmed by using the BLDB database (http://bldb.eu/).^30^ Figures were generated using Easyfig v2.2.2, Proksee (https://proksee.ca/) and compiled using Adobe Photoshop 2022.

## Results

### ICE-PmuST394: a novel ICE in Pasteurella multocida 17BRD-035

**Figure 1.**
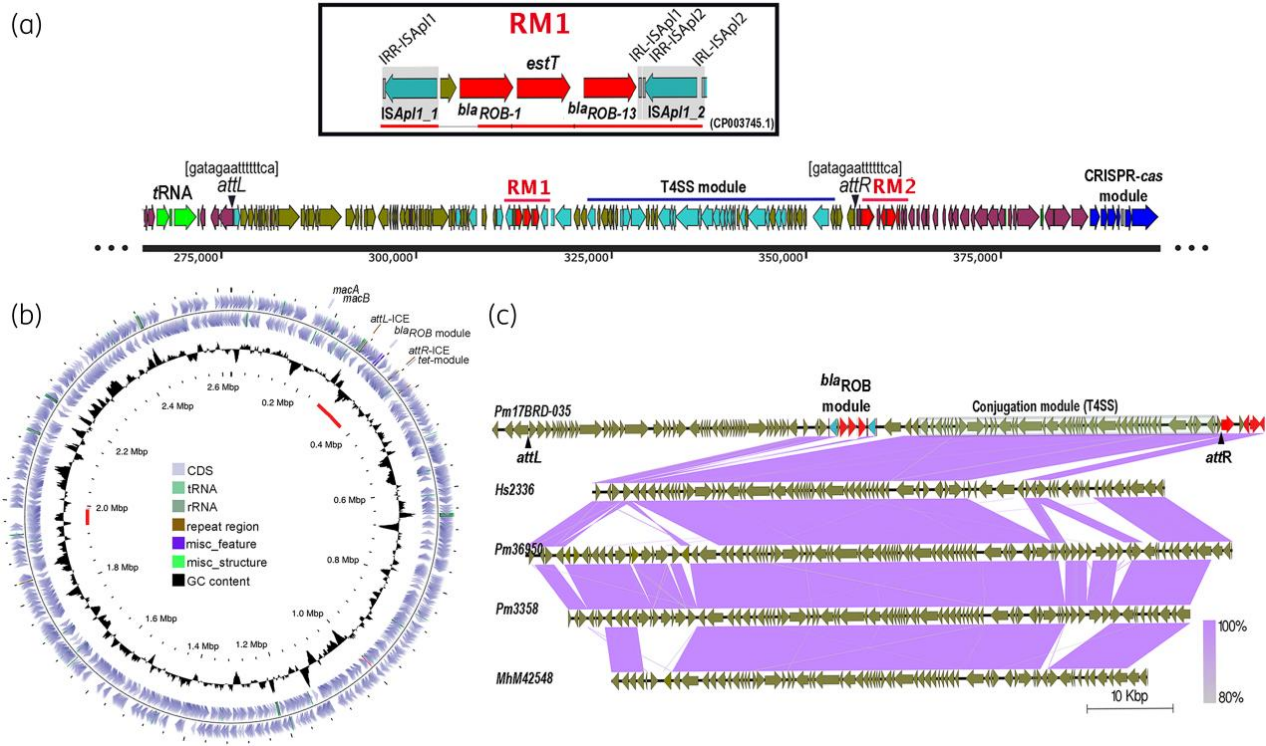
(a) Diagrammatic representation of ICE-*Pmu*ST394 in 17BRD-035. The linear scale indicates the genome coordinates of 17BRD-035 sequence, the ST394 reference genome in GenBank (CP082272.1). Red arrows indicate resistance genes, bottle-green arrows indicate hypothetical genes and aqua-coloured arrows indicate the T4SS-associated conjugation genes. The two resistance modules are labelled as RM1 (*bla*_ROB_-*estT* resistance module) and RM2 [*tet*(R)-*tet*(H) and *mco-ebr*, drug and metal resistance module]. RM1 is detailed in the inset and shows relative organization of *bla*_ROB_ and *estT* resistance genes and the conjugated IS*Apl1* elements demarcating the boundaries of the module. The red bar at the bottom of the inset indicates BLAST alignment of the module with the *B. trehalosi* genome sequence in the GenBank database. (b) Circular map of the *P. multocida* ST394 reference genome, 17BRD-035, with the relative position of ICE-*Pmu*ST394 and the putative IME indicated with red arcs along the innermost circle representing the scale. (c) Alignment of ICE-*Pmu*ST394 with the most closely related ICEs in the ICEberg database: Hs2336 is *H. somnus* 2336 (CP000947), genome coordinates 1 927 921–1 994 529; Pm3690 is *P. multocida* 36950 (CP003022), genome coordinates 273 284–355 497; Pm3358 is *P. multocida* 3358 (CP029712.1), genome coordinates 247 960–320 250; MhM42548 is *M. haemolytica* M42548 (CP005383), genome coordinates 2 128 248–2 190 602. The gradient at the right-hand bottom corner indicates percentage of identity across the genes in the respective genomes. This figure appears in colour in the online version of *JAC* and in black and white in the print version of *JAC*.

Analysis of regions around the resistance genes in 17BRD-035 led to the identification of an 81 546 bp ICE, presented here as ICE-*Pmu*ST394, between coordinates 275 069 bp and 356 614 bp (Figure 1a and b). Average GC content of the region was 39.11%. The boundaries of ICE-*Pmu*ST394 are demarcated by 15 bp (gatagaattttttca) *attL* (275 069–275 083) and *attR* (356 600–356 614) sites, within which lies a full complement of genes required for a functional conjugation module associated with a type IV secretion system (T4SS), two allelic variants of *bla*_ROB_, and the recently identified *estT* resistance gene.^31^ The ICE consists of 102 open reading frames (ORF), including two integrase genes encoding a functional XerC tyrosine recombinase and a third integrase truncated by an IS*Apl1* insertion element (IS*Apl1*_2 in Figure 1a, inset). The ICE also has a relaxase gene, but the *oriT* site could not be predicted. The conjugative module is 99.79% identical to the conjugative module in ICE*Hs*1 from *Histophilus somni* strain AVI 1 (MF136609.1).

The resistance module within ICE-*Pmu*ST394 in *P. multocida* comprises two allelic variants of *bla*_ROB_, *bla*_ROB-1_ and *bla*_ROB-13_, separated by a single ORF encoding the recently characterized serine-dependent macrolide esterase, EstT. It is located within the IR_L_ and the start site of the transposase gene in IS*Apl1* (Figure 1a, inset). Adjacent to the IR_L_ is a second intact copy of the IS*Apl1* insertion element truncating a XerC-like integrase. The 5559 bp resistance module, delineated by two copies of IS*Apl1* (Figure 1a, inset), is located within genome coordinates 311 522–317 080 of the *P. multocida* 17BRD-035 genome and does not appear to have inserted as a single unit. The entire module does not have any match in GenBank; however, in parts, 95% of the module has >99.43% sequence identity with regions of the *Bibersteinia trehalosi* USDA-ARS-USMARC-192 chromosome (CP003745.1) (Figure S1, available as Supplementary data at *JAC* Online). Multiple phage-like integrase genes are present around the module.

A homologue of the macrolide esterase gene *estT*, recently characterized from *Sphingobacterium faecium*,^31^ is located between the two *bla*_ROB_ alleles in ICE-*Pmu*ST394. It is 849 bp in length, which is shorter than the 931 bp *estT* gene characterized in *S. faecium*, and differences in the translated amino acid sequences are localized at the N-terminal end of the peptide sequence (Figure S2). Beyond the N-terminal end, the peptide sequences are 99.6% identical with a single serine to methionine conversion (Figure S2) closer to the C-terminal end. Alignment analysis of *estT* gene with a collection of *P. multocida* genomes^17,18^ led to the identification of five in-house (BRD17-035, 18BRD-001, 19BRD-032, 19BRD-042 and 19BRD-057) and four RefSeq genomes (GCF_001930605.1, GCF_002859385.1, GCF_002859485.1 and GCF_003261475.1), which host the homologue.

The genome of 17BRD-035 additionally had a 46 801 bp putative IME between genome coordinates 1 948 490 and 1 995 290 (Figure 1b). The element had 33 bp (aaaaatgcgagtaattaactcgcattttttgtt) *attL* (1 948 490–1 948 522) and *attR* (1 995 258–1 995 290) sites but did not have a conjugative module. It comprised 63 ORFs, including an integrase gene encoding a XerC tyrosine recombinase, a relaxase and a *mobF* gene. Evidently, the module is immobile but can possibly mobilize if conjugative functions are provided in trans.

### Genetic context of tetracycline and putative macrolide resistance efflux pump in Pasteurella multocida 17BRD-035 (CP082272.1)

The *tet*(R)-*tet*(H) genes were present within coordinates 359 787–361 704 of the *P. multocida* 17BRD-035 genome, but not within the boundaries of ICE-*Pmu*ST394. However, the ICE and other metal and drug resistance genes in 17BRD-035 are housed within a 186 kb (coordinates 175 698 and 361 704) highly plastic genomic region, bounded by multiple *tRNA* genes upstream and the CRISPR-cas-associated genes downstream (Figure 1a); a genomic location well documented to be an insertion site for ICEs in Pasteurellaceae.

An *mco* multicopper oxidase gene resided upstream of the tetracycline resistance module and an *ebrB* gene, known to encode an MDR protein of the SMR family, was found downstream. The combination of genes creates a drug and metal resistance module. The region is 99.9% identical to the chromosome of *H. somni* strain ASc-MMNZ-VFA-069 (CP066739.1) and ICE*Hs*1 (MF136609.1) from *H. somni* strain AVI 1. The CRISPR-cas-associated region is present downstream between genome coordinates 386 534–395 293 bp.

The *macA-macB* genes encoding proteins that constitute a macrolide-specific ABC-type efflux pump in *E. coli*^32^ was present within genome coordinates 175 698–178 771 adjacent to the *tRNA-leu* and *tRNA-cis* genes. We were unable to trace any mobile genetic element in the vicinity of *macA-macB*. The *macA-macB* genes were identified in 130 (of 139) in-house and 177 *P. multocida* genomes in the RefSeq database.

### Comparison of ICE-PmuST394 with closely related ICEs

ICE-*Pmu*ST394 was most closely related to regions of the ICE found in *M. haemolytica* strain M42548 (CP005383, genome coordinates 2 128 248–2 190 602), followed by *Haemophilus somnus* (*H. somni*) strain 2336 (CP000947, genome coordinates 1 927 921–1 994 529) and *P. multocida* strain 36950 (CP003022, genome coordinates 273 284–355 497) (Figure 1c) in the ICEberg database. Alignment of the three ICEs with ICE-*Pmu*ST394 (Figure 1b) indicated a near-identical T4SS module but the *bla*_ROB_-*estT* resistance module was absent. Unlike *P. multocida* 17BRD-035, the tetracycline resistance module in all three related ICEs was positioned within the boundaries of the respective ICE. The ICE found in *P. multocida* strain 3358 is similar to the three ICEs mentioned above (Figure 1c).

### ICE-PmuST394 and its variants within in-house Pasteurella multocida ST394 genomes

**Figure 2.**
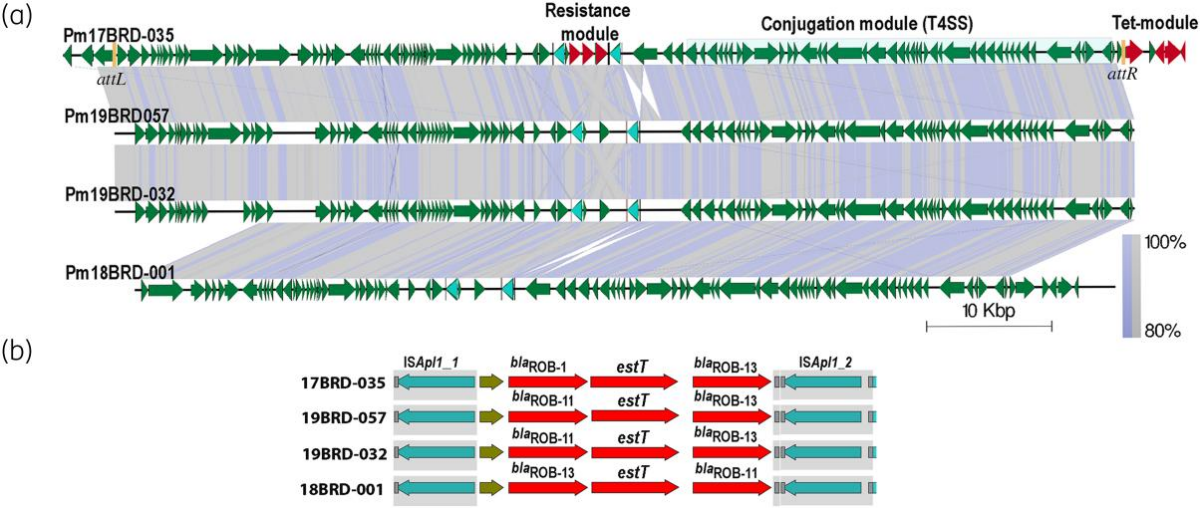
(a) Alignment of ICE-*Pmu*ST394 in 17BRD-035 with ICEs in three other *P. multocida* ST394 genomes in our collection: 19BRD-057, 19BRD-032 and 18BRD-001. Boundaries (*attL* and *attR*) of ICE-*Pmu*ST394 are demarcated by yellow bars in the diagram. The gradient at the right-hand corner indicates percentage of identity across the genes in the respective genomes. (b) Details of the *bla*_ROB_ resistance module in ICEs of the four ST394 genomes in our collection. This figure appears in colour in the online version of *JAC* and in black and white in the print version of *JAC*.

ST394 isolates 18BRD-001, 19BRD-032 and 19BRD-057 in our collection displayed identical resistance profiles to 17BRD-035. Of these three, the genome of 19BRD-057 was completely closed while the other two are draft genomes. All three genomes had minor variants of ICE-*Pmu*ST394, with >99.9% sequence similarity across their lengths (Figure 2a). In isolate 19BRD-057, the ICE was located within genome coordinates 275 029 and 356 573, while in isolate 19BRD-032, the element was present between genome coordinates 1 152 646 and 1 234 190. Both had identical *attL* and *attR* sites and the length of the ICE was identical to ICE-*Pmu*ST394. However, in isolate 18BRD-001 the ICE was shorter (78 463 bp) and the sequence of the *attL* and *attR* sites (tgggatttttggcgt) was different, indicating a separate integration site and consequently non-clonal dissemination of ICE-*Pmu*ST394.

Alignment of ICE-*Pmu*ST394 with the ICEs in three other ST394 genomes using TBLASTX revealed differences in the *bla*_ROB_ alleles and in the ORFs abutting the resistance module (Figure 2a and b). Isolates 19BRD-032 and 19BRD-057 had *bla*_ROB-11_ and *bla*_ROB-13_ alleles, while 17BRD-035 and 18BRD-001 had *bla*_ROB-1_ and *bla*_ROB-13_, and *bla*_ROB-13_ and *bla*_ROB-11_ (Figure 2b), respectively. The fragment containing the truncated integrase gene IS*Apl1*_2 in (Figure 1a, inset) and the 1980 bp *traI* homologue adjacent to it in isolate 17BRD-035, is inverted in 19BRD-057 and 19BRD-032. In addition, there are several differences in the hypothetical gene cargo of the four isolates (Figure 2a). However, the conjugation module is identical in all ICE variants.

### Is ICE-PmuST394 restricted to Australian Pasteurella multocida ST394 strains?

To ascertain the relative abundance of ICE-*Pmu*ST394, and its characteristic modules, in a global collection of *P. multocida* genomes, we searched 342 *P. multocida* genomes including 139 in-house Australian *P. multocida* genomes. We used three different query sequences and filtered results using stringent cut-off parameters. The first query sequence used was the complete sequence (81 546 bp) of ICE-*Pmu*ST394. Given the size of the query, only genomes that returned >97.5% identity over >20 kb alignment lengths were selected as positive hits (column 1 of Table S1). The entire conjugation module (38 874 bp) was used as a query in the second search and >97.5% identity over 100% query length was used to select positive hits (column 2, Table S1). The third query included all three allelic variants of *bla*_ROB_ found on the β*-*lactamase resistance modules identified here, where >99% identity over 100% query length was used to score a positive hit (column 3, Table S1). The conjugative module and the *bla*_ROB_ genes co-reside in five isolates (BRD17-035, 18BRD-001, 19BRD-032, 19BRD-042 and 19BRD-057) in our in-house collection and one isolate from the RefSeq database (GCF_001930605.1). Except for isolate 19BRD-042, all in-house isolates were representatives of RIRDC MLST ST394. 19BRD-042 belongs to ST125 and is a fragmented genome assembled from Illumina short-read sequences. However, alignment of ICE-*Pmu*ST394 with the 19BRD-042 genome sequence indicates 100% coverage of the ICE. Interrogation of the genome sequence GCF_001930605.1 (*P. multocida* strain 2887PM) returned a positive hit for all modules. RIRDC MLST typing of *P. multocida* strain 2887PM indicated it was a representative of ST79 and was cultured from a nasopharyngeal swab of a dairy cow in California, USA. The genome was fragmented; however, contig 11 contained the most significant match to ICE-*Pmu*ST394. Beyond the conjugative module, there were several fragmented hits. However, the characteristic β*-*lactamase/macrolide resistance module of ICE-*Pmu*ST394 was absent in the genome.

*bla*_ROB-1_ has previously been reported to be present adjacent to a fragmented IS*Apl*1 on plasmid pB1000 from *Haemophilus parasuis* strain BB1021 (DQ840517.2). The sequence of all ORFs on pB1000 was used to test association, if any, of the IS*Apl1* on the plasmid with a *bla*_ROB_-containing module on ICE-*Pmu*ST394 and with all genomes in our collection. Our analysis indicates that none of the *P. multocida* genomes host pB1000-like plasmids except isolate GCF_001930605.1.

## Discussion

The distinguishing features of an 81 546 bp ICE, ICE-*Pmu*ST394, identified on the genome of *P. multocida* 17BRD-035, a reference genome for *P. multocida* ST394 from Australia, are presented here. The ICE has a unique resistance module comprising two allelic variants of the β*-*lactamase-encoding *bla*_ROB_ gene (*bla*_ROB-1_ and *bla*_ROB-13_) and the serine-dependent macrolide esterase gene *estT*, delineated by two copies of IS*Apl1*. The conjugation module is near identical to ICE*Mh1* in *M. haemolytica* strain 42548,^9^ ICE in *H. somni* strain 2336,^33^ ICE*Pmu*1 on *P. multocida* strain 36950^10^ and ICE*Pmu*3358,^8^ but the resistance gene load is relatively low when compared to the other region-specific ICEs in the Pasteurellaceae. This element has 102 ORFs, including 2 functional tyrosine recombinase-type integrase genes, and a relaxase. An ICE-*Pmu*ST394-like element was also identified on a non-ST394 *P. multocida* genome, 19BRD-042 (ST125) in our collection. Furthermore, using a global collection of *P. multocida* genomes downloaded from the GenBank RefSeq database, we present convincing evidence that ICE-*Pmu*ST394 is mostly restricted to Australian *P. multocida* isolates, except the one possible ST79 isolate from the USA. Variants of ICE-*Pmu*ST394 were identified on three other ST394 genomes in our collection: 19BRD-057, 19BRD-032 and 18BRD-001. The 5560 bp resistance module has different variants of the *bla*_ROB_ allele: *bla*_ROB-1_/*bla*_ROB-13_ in 17BRD-035, and *bla*_ROB-13_/*bla*_ROB-11_ in 18BRD-001. Our analysis suggests that these variants are likely a reflection of mutational changes in *bla*_ROB_, highlighting ongoing micro-evolutionary events occurring locally.

At a nucleotide level the *bla*_ROB-1_ allele differs from *bla*_ROB-13_ by three bases, which translates to a difference of three amino acids in the peptide sequence. In comparison, *bla*_ROB-1_ differs from *bla*_ROB-11_ by one nucleotide and a single amino acid change. Therefore, it is likely that the *bla*_ROB-11_ seen in 2019 isolates (19BRD-032 and 19BRD-057) were a result of a single mutation in *bla*_ROB-1_ circulating in the 2017 isolate (17BRD-035). The *bla*_ROB-1_ allele was first reported in 1981 from an ampicillin-resistant *Haemophilus influenzae* strain^34^ and was later found in *Haemophilus* (now *Actinobacillus*) *pleuropneumoniae*^35^ plasmids. *bla*_ROB_ encodes a class A β*-*lactamase that is capable of hydrolysing penicillins and first-generation cephalosporins.^36^ By 1988, the gene was reported on plasmids and genomes of *P. multocida* originating from animals.^37^ It is likely that association of IS*Apl1* with *bla*_ROB-1_ has catalysed intragenome mobilization of the resistance gene in *P. multocida*.

The association of IS*Apl1* with *bla*_ROB-1_ has previously been documented on several occasions in *P. multocida* plasmids, including pOV (accession number NC_019381)^38^ and pB1001.^7^ The relative orientation of the ORFs of *bla*_ROB-1_ and the transposase gene in IS*Apl1* were, to the best of our knowledge, always observed in the same orientation. This relative orientation of the IS*Apl1* transposase and *bla*_ROB-1_ genes has recently been reported in ICEHpsaHPS7 from *Glaesserella parasuis*^39^ also. However, in ICE-*Pmu*ST394 and its variants in our in-house collection, orientation of the ORFs are opposite to each other, indicating the role of homologous recombination and gene shuffling events in the formation of the resistance module in ICE-*Pmu*ST394. Our argument is further validated by the fact that direct repeats, generally indicating recent insertions of IS elements, were not traceable at either end of the IS*Apl1* elements in ICE-*Pmu*ST394. This gene arrangement therefore presents a unique opportunity to exploit the inverted orientation as a molecular marker for tracking dispersal of ICE-*Pmu*ST394.

The serine-dependent macrolide esterase gene *estT* from *S. faecium* WB1 is a recently characterized macrolide resistance gene, which when cloned and expressed in *E. coli*, preferentially hydrolyses 16-membered ring-containing macrolides (tylosin, tilmicosin and tildipirosin), but not the 14-member ring macrolides,^31^ and tulathromycin, which consists of a mixture of a 13-membered ring (10%) and a 15-membered ring (90%). The *estT* homologue in ICE-*Pmu*ST394 of *P. multocida* genomes in Australia is shorter by 25 amino acids at the N-terminal end, in addition to the single serine-to-methionine conversion closer to the C-terminus. Isolates with the *estT* gene in our collection exhibited high MICs of both tilmicosin and tylosin, but were susceptible to tildipirosin and tulathromycin (Table 1). The difference in the MIC values of the different macrolide antibiotics tested by Dhindwal *et al.*^31^ with that in *P. multocida* in our study is likely a reflection of the host background. It is notable that Dhindwal *et al.* reported a difference in MIC values of tilmicosin and tildipirosin between clones containing the *estT* gene and the catalytically inactive *estT* (S126A) gene variant. Therefore, the observed differences in susceptibility profiles could also be a reflection of the differences in the peptide sequence of the homologue in *P. multocida*. The unique macrolide resistance profile observed in the subset of *Pasteurella* isolates in Australia is contributed by the *estT* gene, as the gene is only present in isolates with the ICE and the four isolates exhibit identical phenotypes (Table 1). The macrolide-specific ABC efflux pump (*macAB*) has no effect on the resistance profile because identical *macA-macB* genes were found in most of the in-house *P. multocida* isolates (130/139), including isolates susceptible to macrolides.^13^ In Australia, tylosin is not typically used to treat BRD cases, tilmicosin has been largely replaced by the more clinically effective tulathromycin, and tildipirosin is not yet registered.^16^ Therefore, the presence of ICE-*Pmu*ST394 in a handful of isolates from a single feedlot is possibly not yet a dire resistance threat that requires immediate attention, but the likely presence of ICE-*Pmu*ST394 in the ST125 isolate may indicate it is more widespread among Pasteurellaceae than its perceived localization within Australia.

**Table 1.** Antimicrobial susceptibility testing results for four *P. multocida* isolates with ICE-*Pmu*ST394

	FLOR	TET	PEN	TILMIC	TULA	TYLO	TILID
17BRD-035R	0.5	>8	8	32	≤1	16	1
18BRD-001R	0.5	>8	>8	32	≤1	16	1
19BRD-032R	≤0.25	>8	>8	32	≤1	16	1
19BRD-057R	0.5	>8	>8	16 (I)	≤1	16	1
Phenotype	S	R	R	NA^a^	S	NA	S

Numbers indicate MIC (mg/L) corresponding to the tested antimicrobial agents. FLOR, florfenicol; TET, tetracycline; PEN, penicillin; TILMIC, tilmicosin; TULA, tulathromycin; TYLO, tylosin; TILDI, tildipirosin. The last row summarizes phenotypic interpretation for the antibiotics tested: S, sensitive; R, resistant; I, intermediate; NA, veterinary clinical breakpoints are not available.

^a^CLSI tilmicosin veterinary clinical breakpoint for swine is ≥32 mg/L.

Although mobility of ICE-*Pmu*ST394 was not investigated here, we present evidence of ICE-*Pmu*ST394-like elements in at least one ST125 isolate, 19BRD-042. The draft genome was scaffolded, and we were unable to comprehensively characterize the element. However, the sequences of the *attL* and *attR* sites (aaaagtgcggttaaaa) indicate an unrelated integration site compared with ICE-*Pmu*ST394 in 17BRD-035, 19BRD-032 and 19BRD-057, suggesting movement by lateral gene transfer. It is also noteworthy that the sequences of the *attL* and *attR* sites (tgggatttttggcgt) in 18BRD-001 were different to those in 17BRD-035, 19BRD-032 and 19BRD-057. Therefore, it appears that the dispersal of ICE-*Pmu*ST394 in Australian *P. multocida* isolates is being driven by both clonal propagation and lateral movement. Although the resistance gene repertoire on ICE-*Pmu*ST394 is relatively low, the presence of multiple copies of IS*Apl1* can serve as a hotspot for acquisition of additional resistance genes.

### Conclusions

Monitoring the presence and micro-evolution of ICE-*Pmu*ST394 within Pasteurellaceae genomes is necessary to identify evolution of the resistance module and/or variants of the BRD pathogen in Australia. The 5560 bp β*-*lactamase and macrolide resistance module is unique to ICE-*Pmu*ST394 and the relative opposite orientation of the transposase gene in IS*Apl1* and the *bla*_ROB_ allelic variants presents an opportunity to design a trackable molecular marker for targeted surveillance purposes.

## Supplementary Material

dkae040_Supplementary_Data

## Supplementary data

Figures S1, S2 and Table S1 are available as Supplementary data at *JAC* Online.

## References

[1] Griffin D . Economic impact associated with respiratory disease in beef cattle. Vet Clin North Am Food Anim Pract 1997; 13: 367–77. 10.1016/S0749-0720(15)30302-9 9368983

[2] Taylor JD, Fulton RW, Lehenbauer TW et al The epidemiology of bovine respiratory disease: what is the evidence for predisposing factors? Can Vet J 2010; 51: 1095–102. 21197200 PMC2942046

[3] Desmolaize B, Rose S, Wilhelm C et al Combinations of macrolide resistance determinants in field isolates of *Mannheimia haemolytica* and *Pasteurella multocida*. Antimicrob Agents Chemother 2011; 55: 4128–33. 10.1128/AAC.00450-11 21709086 PMC3165316

[4] Kehrenberg C, Catry B, Haesebrouck F et al Tet(L)-mediated tetracycline resistance in bovine *Mannheimia* and *Pasteurella* isolates. J Antimicrob Chemother 2005; 56: 403–6. 10.1093/jac/dki210 15972309

[5] Kehrenberg C, Schulze-Tanzil G, Martel JL et al Antimicrobial resistance in *Pasteurella* and *Mannheimia*: epidemiology and genetic basis. Vet Res 2001; 32: 323–39. 10.1051/vetres:2001128 11432423

[6] Olsen AS, Warrass R, Douthwaite S. Macrolide resistance conferred by rRNA mutations in field isolates of *Mannheimia haemolytica* and *Pasteurella multocida*. J Antimicrob Chemother 2015; 70: 420–3. 10.1093/jac/dku385 25261417

[7] San Millan S, Escudero JA, Gutierrez B et al Multiresistance in *Pasteurella multocida* is mediated by coexistence of small plasmids. Antimicrob Agents Chemother 2009; 53: 3399–404. 10.1128/AAC.01522-08 19528282 PMC2715648

[8] Beker M, Rose S, Lykkebo CA et al Integrative and conjugative elements (ICEs) in Pasteurellaceae species and their detection by multiplex PCR. Front Microbiol 2018; 9: 1329. 10.3389/fmicb.2018.01329 29997583 PMC6028734

[9] Eidam C, Poehlein A, Leimbach A et al Analysis and comparative genomics of ICE*Mh*1, a novel integrative and conjugative element (ICE) of *Mannheimia haemolytica*. J Antimicrob Chemother 2015; 70: 93–7. 10.1093/jac/dku361 25239467

[10] Michael GB, Kadlec K, Sweeney MT et al ICE*Pmu*1, an integrative conjugative element (ICE) of *Pasteurella multocida*: structure and transfer. J Antimicrob Chemother 2012; 67: 91–100. 10.1093/jac/dkr411 22001176

[11] Schink AK, Hanke D, Semmler T et al Novel multiresistance-mediating integrative and conjugative elements carrying unusual antimicrobial resistance genes in *Mannheimia haemolytica* and *Pasteurella multocida*. J Antimicrob Chemother 2022; 77: 2033–5. 10.1093/jac/dkac116 35434742

[12] Meat & Livestock Australia . State of the Industry Report: The Australian red meat and livestock industry 2022-23. 2023. https://www.mla.com.au/globalassets/mla-corporate/prices--markets/documents/trends--analysis/soti-report/mla-state-of-the-industry-report-2223-web_updated.pdf.

[13] Alhamami T, Roy Chowdhury P, Gomes N et al First emergence of resistance to macrolides and tetracycline identified in *Mannheimia haemolytica* and *Pasteurella multocida* isolates from beef feedlots in Australia. Microorganisms 2021; 9:1322. 10.3390/microorganisms9061322 34204544 PMC8233904

[14] Coetzee JF, Cernicchiaro N, Sidhu PK et al Association between antimicrobial drug class selection for treatment and retreatment of bovine respiratory disease and health, performance, and carcass quality outcomes in feedlot cattle. J Anim Sci 2020; 98: skaa109. 10.1093/jas/skaa109 32255182 PMC7179807

[15] Australian Strategic and Technical Advisory Group on Antimicrobial Resistance (ASTAG) . Importance Ratings and Summary of Antibacterial Uses in Human and Animal Health in Australia. 2018; 40. https://www.amr.gov.au/resources/importance-ratings-and-summary-antibacterial-uses-human-and-animal-health-australia.

[16] Badger SM, Sullivan KF, Jordan D et al Antimicrobial use and stewardship practices on Australian beef feedlots. Aust Vet J 2020; 98: 37–47. 10.1111/avj.12889 31721160

[17] Alhamami T, Roy Chowdhury P, Venter H et al Genomic profiling of *Pasteurella multocida* isolated from feedlot cases of bovine respiratory disease. Vet Microbiol 2023; 283: 109773. 10.1016/j.vetmic.2023.109773 37201306

[18] Roy Chowdhury P, Alhamami T, Venter H et al Complete genome sequence of *Pasteurella multocida* sequence type 394, isolated from a case of bovine respiratory disease in Australia. Microbiol Resour Announc 2022; 11: e0089021. 10.1128/mra.00890-21 35234493 PMC8928779

[19] Gaio D, Anantanawat K, To J et al Hackflex: low-cost, high-throughput, Illumina Nextera flex library construction. Microb Genom 2022; 8: 000744. 10.1099/mgen.0.000744 35014949 PMC8914357

[20] Tillett D, Neilan BA. Xanthogenate nucleic acid isolation from cultured and environmental cyanobacteria. J Phycol 2000; 36: 251–8. 10.1046/j.1529-8817.2000.99079.x

[21] Wick RR, Judd LM, Holt KE. Deepbinner: demultiplexing barcoded Oxford Nanopore reads with deep convolutional neural networks. PLoS Comput Biol 2018; 14: e1006583. 10.1371/journal.pcbi.1006583 30458005 PMC6245502

[22] Chen S, Zhou Y, Chen Y et al Fastp: an ultra-fast all-in-one FASTQ preprocessor. Bioinformatics 2018; 34: i884–i90. 10.1093/bioinformatics/bty560 30423086 PMC6129281

[23] Leger A, Leonardi T. pycoQC, interactive quality control for Oxford Nanopore sequencing. J Open Source Softw 2019; 4: 1236–9. 10.21105/joss.01236

[24] Ewels P, Magnusson M, Lundin S et al MultiQC: summarize analysis results for multiple tools and samples in a single report. Bioinformatics 2016; 32: 3047–8. 10.1093/bioinformatics/btw354 27312411 PMC5039924

[25] Wick RR, Judd LM, Gorrie CL et al Unicycler: resolving bacterial genome assemblies from short and long sequencing reads. PLoS Comput Biol 2017; 13: e1005595. 10.1371/journal.pcbi.1005595 28594827 PMC5481147

[26] Seemann T . Prokka: rapid prokaryotic genome annotation. Bioinformatics 2014; 30: 2068–9. 10.1093/bioinformatics/btu153 24642063

[27] Aziz RK, Bartels D, Best AA et al The RAST server: rapid annotations using subsystems technology. BMC Genomics 2008; 9: 75. 10.1186/1471-2164-9-75 18261238 PMC2265698

[28] Liu M, Li X, Xie Y et al ICEberg 2.0: an updated database of bacterial integrative and conjugative elements. Nucleic Acids Res 2019; 47: D660–D5. 10.1093/nar/gky1123 30407568 PMC6323972

[29] Altschul SF, Gish W, Miller W et al Basic local alignment search tool. J Mol Biol 1990; 215: 403–10. 10.1016/S0022-2836(05)80360-2 2231712

[30] Naas T, Oueslati S, Bonnin RA et al Beta-lactamase database (BLDB) - structure and function. J Enzyme Inhib Med Chem 2017; 32: 917–9. 10.1080/14756366.2017.1344235 28719998 PMC6445328

[31] Dhindwal P, Thompson C, Kos D et al A neglected and emerging antimicrobial resistance gene encodes for a serine-dependent macrolide esterase. Proc Natl Acad Sci U S A 2023; 120: e2219827120. 10.1073/pnas.2219827120 PMC9974460 36791107

[32] Kobayashi N, Nishino K, Yamaguchi A. Novel macrolide-specific ABC-type efflux transporter in *Escherichia coli*. J Bacteriol 2001; 183: 5639–44. 10.1128/JB.183.19.5639-5644.2001 11544226 PMC95455

[33] Mohd-Zain Z, Turner SL, Cerdeno-Tarraga AM et al Transferable antibiotic resistance elements in *Haemophilus influenzae* share a common evolutionary origin with a diverse family of syntenic genomic islands. J Bacteriol 2004; 186: 8114–22. 10.1128/JB.186.23.8114-8122.2004 15547285 PMC529066

[34] Rubin LG, Yolken RH, Medeiros AA et al Ampicillin treatment failure of apparently β-lactamase-negative *Haemophilus influenzae* type b meningitis due to novel β-lactamase. Lancet 1981; 2: 1008–10. 10.1016/S0140-6736(81)91214-9 6118476

[35] Hirsh DC, Martin LD Libal MC. Plasmid-mediated antimicrobial resistance in *Haemophilus pleuropneumoniae*. Am J Vet Res 1982; 43: 269–72. 7046533

[36] Medeiros AA, Levesque R, Jacoby GA. An animal source for the ROB-1 beta-lactamase of *Haemophilus influenzae* type b. Antimicrob Agents Chemother 1986; 29: 212–5. 10.1128/AAC.29.2.212 3487284 PMC176379

[37] Livrelli VO, Darfeuille-Richaud A, Rich CD et al Genetic determinant of the ROB-1 beta-lactamase in bovine and porcine *Pasteurella* strains. Antimicrob Agents Chemother 1988; 32: 1282–4. 10.1128/AAC.32.8.1282 3263836 PMC172395

[38] Lopez-Ochoa AJ, Sanchez-Alonso P, Vazquez-Cruz C et al Molecular and genetic characterization of the pOV plasmid from *Pasteurella multocida* and construction of an integration vector for *Gallibacterium anatis*. Plasmid 2019; 103: 45–52. 10.1016/j.plasmid.2019.04.003 31022414

[39] An J, Guo G, Yu D et al ICE*Hpsa*HPS7, a novel multiple drug resistance integrative conjugative element in *Glaesserella parasuis*. Antimicrob Agents Chemother 2021; 65: e01716-20. 10.1128/AAC.01716-20 33199394 PMC7848986

