# Supplementary material for: Identification and evolution of ICE-PmuST394: a novel integrative conjugative element in Pasteurella multocida ST394
Source: J Antimicrob Chemother. 2024 Feb 21;79(4):851–8. doi: 10.1093/jac/dkae040 (PMC10984947; doi:10.1093/jac/dkae040)
Supplement: dkae040_Supplementary_Data [file dkae040_supplementary_data.zip › Supplementary_Figures_S1-S2.docx]

**Figure S1:** Alignment of “Resistance module 1” with genome sequence of *Bibersteinia trehalosi* USDA-ARS-USMARC-192 chromosome (CP003745.1). Coloured arrows represent a diagrammatic representation of ICE-*Pmu*ST394 in 17BRD-035. The red lines indicate regions of ICE-*Pmu*ST394 that align to different sections of the resistance module. Individual fragments of the *B. trehalosi* genome (represented as red lines) are labelled with genomic co-ordinates at both ends of the line and identity across individual fragment length is written in blue text above it.

**
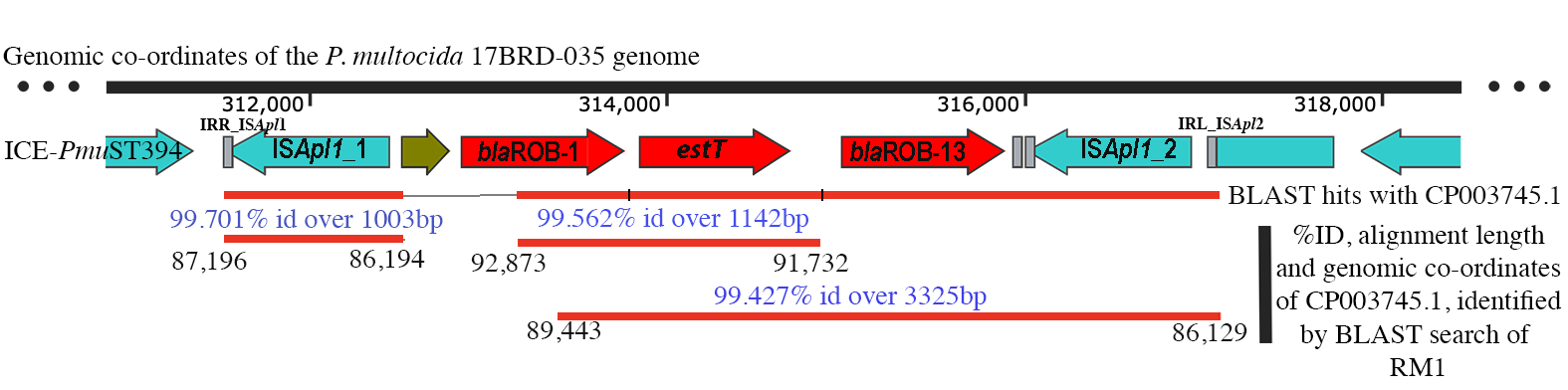
**

**Figure S2:** Alignment of the peptide sequence of EstT homologue in *Sphingobacterium faecium* WB1 and *P. multocida* 17BRD-035 using Needle aligner, and EBLOSUM62 matrix, with gap penalty set at 10.0 and Extended penalty set at 0.05.
