# Supplementary material for: Identification and evolution of ICE-PmuST394: a novel integrative conjugative element in Pasteurella multocida ST394
Source: J Antimicrob Chemother. 2024 Feb 21;79(4):851–8. doi: 10.1093/jac/dkae040 (PMC10984947; doi:10.1093/jac/dkae040)
Supplement: dkae040_Supplementary_Data [file dkae040_supplementary_data.zip › Table S1.docx]

| **ICE-*Pm*ST394 >97.5% ID over 20k bp** | **Conjugative module >99% ID** | ***bla*_ROB_ alelles >99% ID** |
| --- | --- | --- |
|  |  |  |
| *ACARE016.contigs* | *ACARE016.contigs* | *ACARE016.contigs* |
| *ACARE047.contigs* | *ACARE047.contigs* | *ACARE047.contigs* |
| *ACARE091.contigs* | *ACARE091.contigs* | ACARE048.contigs |
| *ACARE097.contigs* | *ACARE097.contigs* | *ACARE091.contigs* |
| *ACARE100.contigs* | *ACARE100.contigs* | *ACARE097.contigs* |
| GCF_000234745.1_ASM23474v1_genomic | GCF_000234745.1_ASM23474v1_genomic | *ACARE100.contigs* |
| GCF_001929465.1_ASM192946v1_genomic | GCF_001929465.1_ASM192946v1_genomic | *GCF_001930605.1_ASM193060v1_genomic* |
| GCF_001929525.1_ASM192952v1_genomic | GCF_001929525.1_ASM192952v1_genomic |  |
| GCF_001929565.1_ASM192956v1_genomic | GCF_001929565.1_ASM192956v1_genomic |  |
| GCF_001929595.1_ASM192959v1_genomic | GCF_001929595.1_ASM192959v1_genomic |  |
| GCF_001929655.1_ASM192965v1_genomic | GCF_001929655.1_ASM192965v1_genomic |  |
| GCF_001929705.1_ASM192970v1_genomic | GCF_001929705.1_ASM192970v1_genomic |  |
| GCF_001929765.1_ASM192976v1_genomic | GCF_001929765.1_ASM192976v1_genomic |  |
| GCF_001929785.1_ASM192978v1_genomic | GCF_001929785.1_ASM192978v1_genomic |  |
| GCF_001929855.1_ASM192985v1_genomic | GCF_001929855.1_ASM192985v1_genomic |  |
| GCF_001929905.1_ASM192990v1_genomic | GCF_001929905.1_ASM192990v1_genomic |  |
| GCF_001929985.1_ASM192998v1_genomic | GCF_001929985.1_ASM192998v1_genomic |  |
| GCF_001930065.1_ASM193006v1_genomic | GCF_001930065.1_ASM193006v1_genomic |  |
| GCF_001930115.1_ASM193011v1_genomic | GCF_001930115.1_ASM193011v1_genomic |  |
| GCF_001930285.1_ASM193028v1_genomic | GCF_001930285.1_ASM193028v1_genomic |  |
| GCF_001930305.1_ASM193030v1_genomic | GCF_001930305.1_ASM193030v1_genomic |  |
| GCF_001930385.1_ASM193038v1_genomic | GCF_001930385.1_ASM193038v1_genomic |  |
| GCF_001930405.1_ASM193040v1_genomic | GCF_001930405.1_ASM193040v1_genomic |  |
| GCF_001930445.1_ASM193044v1_genomic | GCF_001930445.1_ASM193044v1_genomic |  |
| GCF_001930465.1_ASM193046v1_genomic | GCF_001930465.1_ASM193046v1_genomic |  |
| GCF_001930525.1_ASM193052v1_genomic | GCF_001930525.1_ASM193052v1_genomic |  |
| *GCF_001930605.1_ASM193060v1_genomic* | *GCF_001930605.1_ASM193060v1_genomic* |  |
| GCF_001930705.1_ASM193070v1_genomic | GCF_001930705.1_ASM193070v1_genomic |  |
| GCF_001930765.1_ASM193076v1_genomic | GCF_001930765.1_ASM193076v1_genomic |  |
| GCF_001930875.1_ASM193087v1_genomic | GCF_001930875.1_ASM193087v1_genomic |  |
| GCF_001930895.1_ASM193089v1_genomic | GCF_001930895.1_ASM193089v1_genomic |  |
| GCF_001930925.1_ASM193092v1_genomic | GCF_001930925.1_ASM193092v1_genomic |  |
| GCF_001930965.1_ASM193096v1_genomic | GCF_001930965.1_ASM193096v1_genomic |  |
| GCF_001931145.1_ASM193114v1_genomic | GCF_001931145.1_ASM193114v1_genomic |  |
| GCF_001931225.1_ASM193122v1_genomic | GCF_001931225.1_ASM193122v1_genomic |  |
| GCF_001931235.1_ASM193123v1_genomic | GCF_001931235.1_ASM193123v1_genomic |  |
| GCF_001931265.1_ASM193126v1_genomic | GCF_001931265.1_ASM193126v1_genomic |  |
| GCF_002859245.1_ASM285924v1_genomic | GCF_002859245.1_ASM285924v1_genomic |  |
| GCF_002859285.1_ASM285928v1_genomic | GCF_002859285.1_ASM285928v1_genomic |  |
| GCF_002859305.1_ASM285930v1_genomic | GCF_002859305.1_ASM285930v1_genomic |  |
| GCF_002859345.1_ASM285934v1_genomic | GCF_002859345.1_ASM285934v1_genomic |  |
| GCF_002859365.1_ASM285936v1_genomic | GCF_002859365.1_ASM285936v1_genomic |  |
| GCF_002859385.1_ASM285938v1_genomic | GCF_002859385.1_ASM285938v1_genomic |  |
| GCF_002859405.1_ASM285940v1_genomic | GCF_002859405.1_ASM285940v1_genomic |  |
| GCF_002859425.1_ASM285942v1_genomic | GCF_002859425.1_ASM285942v1_genomic |  |
| GCF_002859485.1_ASM285948v1_genomic | GCF_002859485.1_ASM285948v1_genomic |  |
| GCF_002859545.1_ASM285954v1_genomic | GCF_002859545.1_ASM285954v1_genomic |  |
| GCF_003261475.1_ASM326147v1_genomic | GCF_003261475.1_ASM326147v1_genomic |  |
| GCF_009646155.1_ASM964615v1_genomic | GCF_009646155.1_ASM964615v1_genomic |  |

**Table S1:** Summary of BLAST search using various segments of ICE-PmuST394 and a global collection of Pasteurella multocida genomes. The GCF numbers indicate the RefSeq accession numbers and the ASM numbers indicate the Assembly numbers in GenBank database. We also included 139 inhouse P. multocida genomes, designated here with ACARE numbers. Genomes corresponding to the ACARE numbers and their Genbank accession numbers are as follows: ACARE016= genome of isolate 17BRD-035 (JANIEN000000000.1); ACARE047 = genome of isolate 18BRD-001 (JANCXB000000000); ACARE048 = genome of isolate 18BRD-005 (JANCXA000000000) ; ACARE091 = genome of isolate 19BRD-032 (JANCVL000000000); ACARE097 = genome of isolate 19BRD-042 (JANCVF000000000); and ACARE100 = genome of isolate 19BRD-057 (JANCVC000000000). The genomes italicised in all three columns indicate isolates which returned a positive BLAST hit in all three searches.
